# Association between marriage and outcomes in patients with acute ischemic stroke

**DOI:** 10.1007/s00415-018-8793-z

**Published:** 2018-02-20

**Authors:** Qi Liu, Xianwei Wang, Yilong Wang, Chunxue Wang, Xingquan Zhao, Liping Liu, Zixiao Li, Xia Meng, Li Guo, Yongjun Wang

**Affiliations:** 1 Department of Neurology, The Second Hospital of Hebei Medical University, No.215 Hepingxilu, Xinhua District, Shijiazhuang, 050000 Hebei China; 2 Department of Neurology, Beijing Tiantan Hospital, Capital Medical University, No.6 Tiantanxili, Dongcheng District, Beijing, 100050 China; 3 China National Clinical Research Center for Neurological Diseases, Beijing, 100050 China; 4 Center of Stroke, Beijing Institute for Brain Disorders, Beijing, 100050 China; 5 Beijing Key Laboratory of Translational Medicine for Cerebrovascular Disease, Beijing, 100050 China

**Keywords:** Acute ischemic stroke, Marital status, All-cause death, Stroke recurrence, Stroke disability

## Abstract

**Backgrounds:**

The previous studies on the association between marital status and stroke outcomes were rare. Furthermore, the existing studies mostly focused on the protective effect of marriage on survival. We conducted the study to evaluate the association between marital status and adverse stroke outcomes in patients with AIS based on China national stroke registry.

**Methods:**

This was a multicenter, prospective cohort study of patients with AIS. Patients were classified into two groups based on marital status at admission: married and unmarried. The primary outcomes included all-cause mortality, stroke recurrence, combined endpoint, and stroke disability. Stroke disability was defined as modified Rankin Scale of 2–6.

**Results:**

Of 12,118 patients, 1220 were unmarried and 10,898 married. Unmarried patients had higher proportion of 1-year post-stroke events than married patients did. As compared with being unmarried, the adjusted odds ratios with 95% confidence interval of being married for outcomes were as follows: 0.70 (0.58–0.84) for all-cause mortality, 0.78 (0.66–0.91) for stroke recurrence, 0.77 (0.66–0.90) for combined endpoint, and 0.75 (0.65–0.88) for stroke disability. Interactions between marital status and education were significant for all outcomes except for stroke disability.

**Conclusions:**

Marital status was associated with all adverse stroke outcomes in patients with acute ischemic stroke, especially in those with middle-school education.

**Electronic supplementary material:**

The online version of this article (10.1007/s00415-018-8793-z) contains supplementary material, which is available to authorized users.

## Introduction

Stroke is the leading cause of death and adult disability in the worldwide [1, 2]. China has more than 2.5 million new stroke cases each year, and ischemic stroke accounts for 43–79% of all strokes [3]. Controlling risk factors would contribute to stroke prevention and improving survival outcomes. Studies demonstrated that marriage was negatively associated with adverse cardiovascular events [4] and was an independent predictor of survival [5, 6].

Stroke patients might need more social or family supports due to stroke disability. Existing literatures show that unmarried patients are more likely to die following a stroke than married patients [7, 8]. However, there is little data on impact of marriage on recurrent stroke and poor functional outcome, especially among Asian population. We hypothesized that marriage would a protective effect on adverse outcomes in patients with acute ischemic stroke (AIS). Thus, the study aimed to evaluate the association between marital status and stroke outcomes including all-cause mortality, stroke recurrence, and stroke disability based on the China National Stroke Registry (CNSR).

## Methods

### Study population

All study subjects were from a nationwide CNSR which was designed to evaluate the quality of care for hospitalized stroke patients and measure the clinical and functional outcomes at 1 year after disease onset. Details about study rational, design, and results have been published previously [9]. For the present study, subjects were patients who met the following criteria: (1) older than 18; (2) diagnosis of AIS within 14 days after the onset of symptoms, and which are confirmed by brain computed tomography or magnetic resonance imaging; and (3) written informed consent obtained from patients or their legally authorized representatives. From September 2007 to August 2008, 12,415 consecutive patients were included, 233 (1.9%) patients were excluded for missing 1-year follow-up, and 64 (0.5%) were excluded for missing marital status data. Finally, 12,118 (97.6%) patients were analyzed into the current study. (Supplemental figure 1) The study was approved by the central institutional review board at Beijing Tiantan Hospital.

### Data collection

Data collection was completed by trained research coordinators according to a standard protocol. The baseline data extracted from the medical records included patient demographics, socioeconomic status, medical conditions, NIHSS (National Institutes of Health Stroke Scale), and other vascular risk factors. The medical conditions included history of stroke, hypertension, diabetes, dyslipidaemia, atrial fibrillation, coronary heart disease, and pneumonia. Other vascular risk factors included current or previous smoking and moderate or heavy alcohol consumption (≥ 2 standardized alcohol drinks per day) and body mass index (BMI). Socioeconomic status (SES) included educational level, occupational class, and personal income level [10]. Occupational class was classified based on the main job types at admission. Personal income refers average family income per capita per month (e.g., the family’s actual income per month is divided by the number of family members).

### Marital status grouping

Marital status included married, never married, remarried, widowed, and divorced. In the current study, all subjects were divided into two categories: married and unmarried. Considering the small sample of remarried (0.21%), never married (1.13%), and divorced (0.58%), being married included married and remarried while being unmarried included widowed, divorced, and never married [5].

### Outcome assessment

One-year telephone follow-up was conducted by trained research personnel who was blinded to patient’s baseline characteristics. Outcomes included all-cause mortality, stroke recurrence, combined endpoint (death and stroke recurrence), and stroke disability. Stroke recurrence contained ischemic stroke, intracranial hemorrhage, and subarachnoid hemorrhage. During the follow-up periods, stroke recurrence associated with rehospitalization was sourced to the attended hospitals to ensure a reliable diagnosis. In the case of a suspected recurrent cerebrovascular event without hospitalization, judgement was made by the research coordinators together with the principal investigator [9]. Stroke disability was defined as modified Rankin Scale of 2–6 [mRS, score ranges from 0 (no symptoms) to 6 (death)].

### Statistical analysis

We compared baseline and clinical characteristics of patients grouped by marital status by *t* test and *χ*^2^ or Fisher’s exact test. Continuous variables were expressed as median (inter-quartile range, IQR), whereas categorical data were presented as proportions.

Multivariable logistic regression model was performed to assess the association between marital status and stroke outcomes with the unmarried group as the reference. The multivariable analysis adjusted the following covariates which were thought to be associated with adverse outcomes: age, sex, region, types of health insurance, history of stroke, hypertension, diabetes, dyslipidaemia, atrial fibrillation, coronary heart disease, pneumonia, current or previous smoking, moderate or heavy alcohol, body mass index (BMI) at admission, baseline NIHSS, living status, and SES. Odds ratios (ORs) with 95% confidence intervals (CIs) were calculated.

We also performed stratification analyses by age (≤ 65 and > 65 years), education (elementary school or below, middle school, and high school or above), gender (male and female), and region (eastern, central and western). To examine effect modification by age, education, gender, and region, we used a post-estimation Wald test in multivariable-adjusted logistic model to get an omnibus *P* value for interaction between marital status categories and variables of interest.

A two-sided *P* value < 0.05 was set as the level for statistical significance. All analyses were performed with SAS software version 9.3 (SAS Institute Inc, Cary, NC, USA).

## Results

### Study population and characteristics

Of the final 12,118 patients in this study, 1220 (10.1%) were unmarried and 10,898 (89.9%) married (Supplementary Fig. 1). The median age was 67 years. As compared with married, unmarried patients were older, more likely to be female, to have lower BMI at baseline, to live alone, and to have elementary or below education, to be non-working status (retired or no job). In addition, unmarried patients were more likely to have severe stroke, to have a history of coronary heart disease, atrial fibrillation, and pneumonia. Conversely, they were less likely to be smoker and heavy drinker, to live in eastern region, and to have diabetes mellitus and hyperlipidemia than married patients did. There was no significant difference in personal income, history of stoke, and hypertension between the two groups (Table 1).

**Table 1:** Baseline characteristics of patients with acute ischemic stroke according to marital status categories

Characteristics	Overall (*n* = 12,118)	Unmarried (*n* = 1220)	Married (*n* = 10,898)	*P* value
Age (years), median (IQR)	67 (57–75)	77 (69–82)	66 (56–74)	< 0.001
Female, no. (%)	4634 (38.2)	749 (61.4)	3885 (35.6)	< 0.001
BMI at admission (kg/m^2^), no. (%)				0.001
< 25	6643 (60.7)	729 (65.5)	5914 (60.2)	
25–30	3713 (33.9)	323 (29.0)	3390 (34.5)	
> 30	582 (5.3)	61 (5.5)	521 (5.3)	
Region, no. (%)				0.036
Eastern	7678 (63.4)	737 (60.4)	6941 (63.7)	
Central	2525 (20.8)	287 (23.5)	2238 (20.5)	
Western	1915 (15.8)	196 (16.1)	1719 (15.8)	
Ever smoking, no. (%)	4826 (39.8)	344 (28.2)	4482 (41.1)	< 0.001
Heavy alcohol, no. (%)	1147 (9.5)	74 (6.1)	1073 (9.8)	< 0.001
Types of health insurance, no. (%)				0.031
BHIS	7293 (61.7)	741 (61.9)	6552 (61.7)	
NCMS	1981 (16.8)	170 (14.2)	1811 (17.1)	
Self-payment	2262 (19.1)	256 (21.4)	2006 (18.9)	
Other	279 (2.4)	30 (2.5)	249 (2.3)	
Medical conditions, no. (%)				
History of stroke	4142 (34.2)	435 (35.7)	3707 (34.0)	0.252
Diabetes mellitus	2609 (21.5)	233 (19.1)	2376 (21.8)	0.029
Hypertension	7748 (63.9)	763 (62.5)	6985 (64.1)	0.283
Hyperlipidemia	1368 (11.3)	96 (7.9)	1272 (11.7)	< 0.001
CHD	1759 (14.5)	246 (20.2)	1513 (13.9)	< 0.001
Atrial fibrillation	897 (7.4)	140 (11.5)	757 (6.9)	< 0.001
Pneumonia	1411 (11.6)	228 (18.7)	1183 (10.9)	< 0.001
Living status, no. (%)				< 0.001
Living alone	428 (3.6)	245 (20.3)	183 (1.7)	
Living with family	11,563 (96.0)	936 (77.7)	10,627 (98.1)	
Nursing home	50 (0.4)	24 (2.0)	26 (0.2)	
Baseline NIHSS group, no. (%)				< 0.001
< 4	10,028 (82.8)	959 (78.7)	9069 (83.2)	
5–14	1664 (13.7)	184 (15.1)	1480 (13.6)	
> 14	422 (3.5)	75 (6.2)	347 (3.2)	
Socioeconomic status				
Educational level, no. (%)				< 0.001
ESB	5535 (45.7)	788 (64.6)	4747 (43.6)	
MS	3138 (25.9)	216 (17.7)	2922 (26.8)	
HSA	3445 (28.4)	216 (17.7)	3229 (29.6)	
Occupational class, no. (%)				< 0.001
Non-manual workers	1598 (13.2)	91 (7.5)	1507 (13.8)	
Manual workers	3282 (27.1)	232 (19.0)	3050 (28.0)	
No job	1278 (10.5)	230 (18.9)	1048 (9.6)	
Retired	5146 (42.5)	598 (49.0)	4548 (41.7)	
Unknown	814 (6.7)	69 (5.7)	745 (6.8)	
Personal income, RMB/mon, no. (%)				0.092
≤ 1000	4165 (34.4)	447 (36.6)	3718 (34.1)	
1000–3000	4081 (33.7)	420 (34.4)	3661 (33.6)	
300–5000	649 (5.4)	50 (4.1)	599 (5.5)	
> 5000	153 (1.3)	13 (1.1)	140 (1.3)	
Unknown	3070 (25.3)	290 (23.8)	2780 (25.5)	

*IQR* inter-quartile range, *BMI* body mass index, *ESB* elementary school or below, *MS* middle school, HSA high school or above, *BHIS* Basic Health Insurance Scheme for urban and governmental, *NCMS* New Cooperative Medical System, *CHD* coronary heart disease, *NIHSS* National Institutes of Health Stroke Scale

### One-year incidence of stroke outcomes

**Fig. 1:**
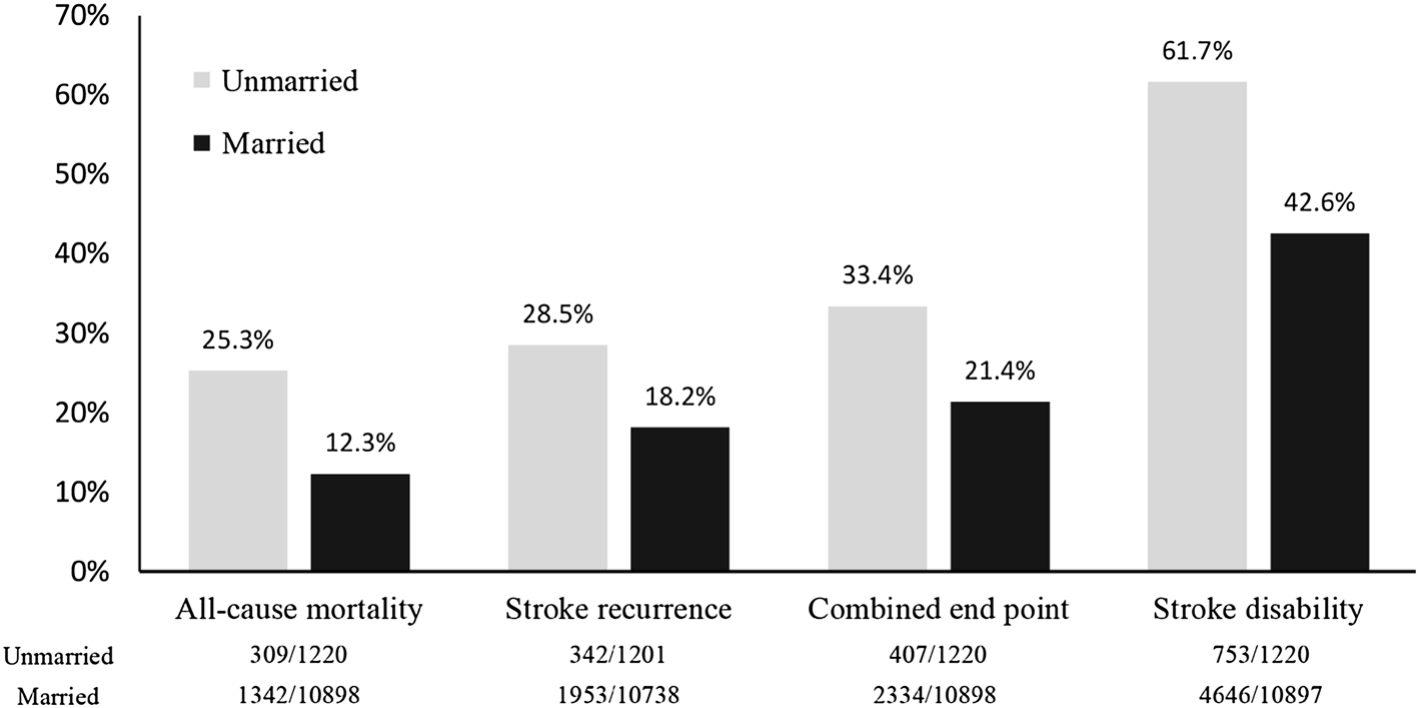
Proportions of 1-year stroke outcomes according to marital status

Unmarried patients had higher proportion of 1-year post-stroke events than married patients: 25.3 versus 12.3% for all-cause mortality (*P* < 0.01), 28.5 versus 18.2% for stroke recurrence (*P* < 0.01), 33.4 versus 21.4% for combined endpoint (*P* < 0.01), and 61.7 versus 42.6% for stroke disability (*P* < 0.01), respectively (Fig. 1).

### Association between marital status and stroke outcomes

**Fig. 2:**
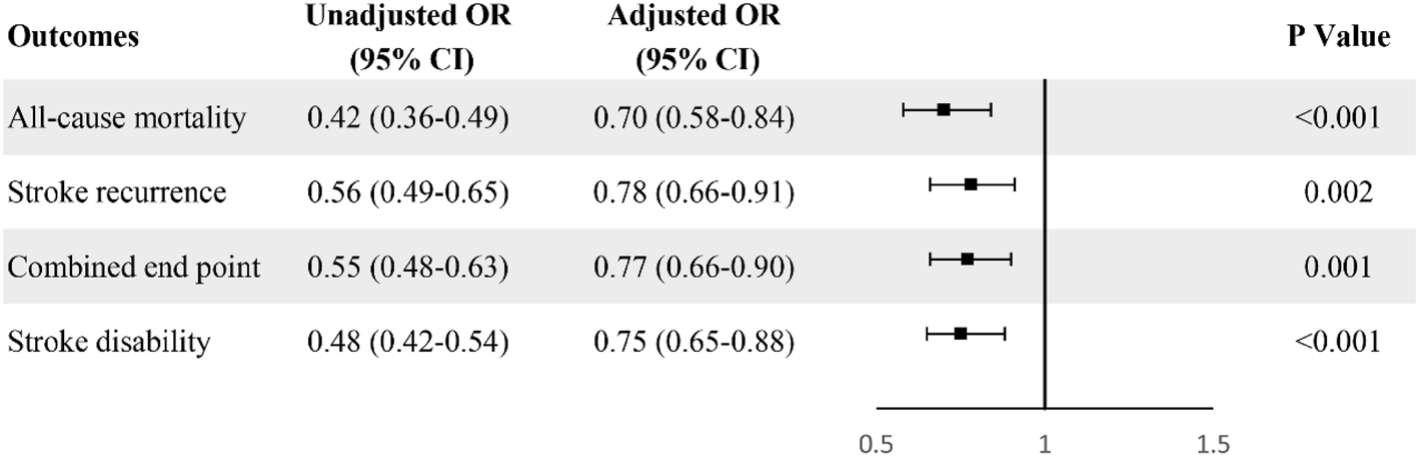
Odds ratios of being married versus being unmarried for all-cause mortality, stroke recurrence, combined end point, and stroke disability. *OR* odds ratio, *CI* confidence interval. Covariates included age, sex, region, types of health insurance, history of stroke, hypertension, diabetes, dyslipidaemia, atrial fibrillation, coronary heart disease, pneumonia, current or previous smoking, moderate or heavy alcohol, body mass index (BMI) at admission, baseline NIHSS, living arrangements, and socioeconomic status (educational level, occupational class, and personal income level)

ORs with 95% CI of marital status for stroke outcomes were reported in Fig. 2. Univariate and multivariate analyses showed that marital status was independently associated with stroke outcomes. As compared with unmarried, the adjusted ORs of married patients were 0.70 (95% CI 0.58–0.84) for all-cause mortality, 0.78 (95% CI 0.66–0.91) for stroke recurrence, 0.77 (95% CI 0.66–0.90) for combined end point, and 0.75 (95% CI 0.65–0.88) for stroke disability, respectively.

ORs and 95% CI of marital status for stroke outcomes in subjects stratified by age, gender, region, and education were shown in Table 2. We found that marriage was independently associated with all stroke outcomes among patients with the middle-school education: 0.43 (95% CI 0.29–0.65) for all-cause mortality, 0.54 (95% CI 0.38–0.77) for stroke recurrence, 0.52 (95% CI 0.37–0.72) for combined endpoint, and 0.60 (95% CI 0.43–0.83) for stroke disability. In addition, significant interactions between marriage and education for all-cause mortality (*P* = 0.047) and combined endpoint (*P* = 0.05) were also identified, but not for stroke recurrence (*P* = 0.07) and stroke disability (*P* = 0.35). There were no significant interactions between marriage status and age, gender, or region for all stroke outcomes.

**Table 2:** Adjusted odds ratios of married versus unmarried for all-cause mortality, stroke recurrence, combined endpoint, and stroke disability in patients stratified by age, gender, region, and education

	All-cause mortality			Stroke recurrence			Combined end point			Stroke disability		
	OR (95% CI)	*P*	P*	OR (95% CI)	*P*	P*	OR (95% CI)	*P*	P*	OR (95% CI)	*P*	P*
Age			0.29			0.22			0.15			0.33
≤ 65 (years)	0.80 (0.46–1.36)	0.40		0.92 (0.62–1.37)	0.68		0.94 (0.64–1.38)	0.74		0.82 (0.59–1.12)	0.36	
> 65 (years)	0.60 (0.49–0.72)	< 0.01		0.68 (0.58–0.81)	< 0.01		0.68 (0.58–0.81)	< 0.01		0.67 (0.57–0.79)	< 0.01	
Gender			0.18			0.82			0.56			0.25
Male	0.61 (0.46–0.81)	< 0.01		0.80 (0.62–1.02)	0.07		0.73 (0.58–0.93)	< 0.01		0.83 (0.66–1.04)	0.11	
Female	0.78 (0.61–0.99)	0.04		0.75 (0.61–0.93)	< 0.01		0.81 (0.66–0.99)	0.04		0.70 (0.57–0.86)	< 0.01	
Region			0.35			0.99			0.92			0.27
Eastern	0.79 (0.63–1.00)	0.05		0.78 (0.64–0.96)	0.02		0.81 (0.66–0.99)	0.04		0.68 (0.56–0.83)	< 0.01	
Central	0.52 (0.36–0.76)	< 0.01		0.78 (0.56–1.08)	0.14		0.70 (0.51–0.97)	0.03		0.87 (0.64–1.18)	0.36	
Western	0.70 (0.44–1.14)	0.15		0.72 (0.48–1.09)	0.12		0.73 (0.50–1.08)	0.12		0.87 (0.61–1.26)	0.47	
Education			0.047			0.07			0.05			0.35
EOB	0.80 (0.64–1.00)	0.05		0.91 (0.74–1.12)	0.38		0.91 (0.75–1.11)	0.35		0.79 (0.65–0.95)	0.01	
MID	0.43 (0.29–0.65)	< 0.01		0.54 (0.38–0.77)	< 0.01		0.52 (0.37–0.72)	< 0.01		0.60 (0.43–0.83)	< 0.01	
HOA	0.79 (0.49–1.27)	0.33		0.73 (0.51–1.06)	0.10		0.78 (0.55–1.12)	0.17		0.93(0.65–1.32)	0.67	

Adjusted for age (not used in subjects stratified by age), gender (not used in subjects stratified by gender), region (not used in subjects stratified by region), education (not used in subjects stratified by education), types of health insurance, history of stroke, hypertension, dyslipidaemia, pneumonia, atrial fibrillation, coronary heart disease, diabetes, current smoking, heavy alcohol, body mass index at admission, baseline national institutes of health stroke scale, living arrangements, personal income, and occupational class

*ORs* odds ratios, *ESB* elementary school or below, *MS* middle school, *HSA* high school or above

In our study, we did not find the association between living arrangements and post-stroke outcomes among unmarried patients (Supplementary Table 1). In addition, multivariate analysis also showed that the association between marital status and stroke outcomes was similar, regardless of whether occupational class and personal income level were the adjustment factors (Supplementary Table 2).

## Discussion

The current study demonstrated that marital status was independently associated with post-stroke outcomes, especially in patients with middle-school education. The 1-year incidences of stroke outcomes after AIS in unmarried patients were approximately 1.5–2.0 times as high as those in married patients.

To our knowledge, the previous studies on the association between marital status and stroke outcomes were rare. Furthermore, the existing studies mostly focused on the protective effect of marriage on survival. Stroke recurrence and disability as severe adverse outcomes were rarely concerned. A recent study published in *J Am Heart Ass*. found that marital history was significantly associated with survival after stroke. However, this study population was derived from a prospective cohort study of US adults based on patients’ reports of stroke according to interviews rather than precise clinical data [7]. Some other studies on marital status and mortality in elderly based on national population included a systematic review and meta-analysis [6]. Our study assessed the association of marital status and stroke outcomes included all-cause mortality, stroke recurrence, combined endpoint, and stroke disability based on a nationwide prospective cohort study of hospitalized stroke patients. However, a recent study showed that the social isolation rather than marital status was associated with all-cause death after stroke [11]. It was a small study with 655 stroke patients and only 33% subjects were married. In addition, patients who had pre-stroke disability and received help at home were more likely classified into social isolation, which might lead to bias of misclassification.

Studies about the association between living arrangements and stroke outcomes are controversial. The previous studies have showed that patients living alone were more likely to die after stroke episode [12]. However, a recent study found patients living alone had delayed hospital arrival and less thrombolytic therapy, but no significant differences in mortality or readmissions by living status [13]. In the current study, association between living arrangement and stroke outcomes was analyzed only among unmarried patients, since living alone nearly did not happen among married patients. We did not find the association between living status and stroke outcomes in unmarried patients. The plausible explanation would be that living-alone patients might crossover to live with their families for their daily care after stroke, especially patients with moderate-to-severe stroke. The further analysis for this was not made, because we did not collect data on living status during follow-up period.

In our study, stratification analysis showed that effects of marriage on stroke outcomes were significant in patients with middle-school education but not in those with high-or-above education. Furthermore, its interaction for all-cause mortality was significant. We speculated that patient with high school or above education were more likely to have better social support resources, which might partly account for or weaken the protection effect of marriage. In addition, our study found that the association between marital status and stroke outcomes was similar, regardless of whether occupational class and personal income level were the adjustment factors. The previous studies on education related to stroke outcomes were insufficient. A Swedish study found high education and income were associated with a reduced risk of stroke recurrence [14]. A Danish study found education had only a modest effect on survival after stroke episode and only in patients aged < 65 years [15]. Future studies would be necessary to assess the association between education and marriage and their effects on outcomes among patients with stroke.

At present, the prevailing argument about the protective effect of marriage is that marriage might provide more stable behavioral and psychosocial resources to prevent and treat illness [13]. In addition, possible pathophysiological mechanisms related to stress of unmarried have also been reported [16, 17]. Although marital status is not amenable to medical intervention or treatment, understanding the mechanism could help us to identify possible interventions to reduce these risks, and that is the important area for our future research.

Our study also has limitations. First, although we have adjusted many covariates known to be associated with adverse outcomes, there may be residual confounding which potentially influence our results. In addition, we could not evaluate the level of anxiety or depression in unmarried patients in the study, which might have an interaction for the association. Second, the study was performed based on the Chinese stroke patients, so it would be not generalizable to other races or ethics in which cultural discrepancy in marriage might exist. Finally, this was an observational study, so the results did not imply causation of marriage with stroke outcomes.

In conclusion, marriage was independently associated with post-stroke outcomes in patients with acute ischemic stroke, especially in those with middle-school education. Identifying the association could help us to provide the patients with risk awareness related to marital status.

## Electronic supplementary material

Below is the link to the electronic supplementary material.

Supplementary material 1 (DOCX 157 kb)
