# Supplementary material for: Association between marriage and outcomes in patients with acute ischemic stroke
Source: J Neurol. 2018 Feb 20;265(4):942–8. doi: 10.1007/s00415-018-8793-z (PMC5878185; doi:10.1007/s00415-018-8793-z)
Supplement: Supplementary file 1 — Supplementary material 1 (DOCX 157 kb) [file 415_2018_8793_MOESM1_ESM.docx]

**Supplementary Table 1:** Adjusted odds ratios of living alone versus living with family for all stroke outcomes.

| Outcomes | No. of events (%) | |  | Adjusted OR (95% CI) | P |
| --- | --- | --- | --- | --- | --- |
|  | Living alone | Living with family |  |  |  |
| All-cause mortality | 51 (20.8) | 243 (26.0) |  | 0.84 (0.56-1.27) | 0.42 |
|  |  |  |  |  |  |
| Stroke recurrence | 52 (21.8) | 272 (29.5) |  | 1.12 (0.75-1.66) | 0.58 |
|  |  |  |  |  |  |
| Combined end point | 69 (28.2) | 318 (34.0) |  | 0.96 (0.66-1.38) | 0.82 |
|  |  |  |  |  |  |
| Stroke disability | 125 (51.0) | 595 (63.6) |  | 1.16 (0.83-1.64) | 0.39 |
|  |  |  |  |  |  |

OR denotes odds ratios; CI, confidence interval; Adjusted for age, sex, region, education, types of health insurance, history of stroke, hypertension, diabetes, dyslipidaemia, atrial fibrillation, coronary heart disease, pneumonia, current or previous smoking, moderate or heavy alcohol, body mass index (BMI) at admission and baseline NIHSS.

**Supplementary Table 2:** Odds ratios for stroke outcomes associated with marital status.

| **Outcomes** | **Adjusted OR （95% CI）** | |
| --- | --- | --- |
|  | **Model 1** | **Model 2** |
| All-cause mortality | 0.71 (0.59-0.85) | 0.70 (0.58-0.84) |
| Stroke recurrence | 0.78 (0.66-0.92) | 0.78 (0.66-0.91) |
| Combined end point | 0.78 (0.67-0.91) | 0.77 (0.66-0.90) |
| Stroke disability | 0.76 (0.65-0.88) | 0.75 (0.65-0.88) |

The reference group is unmarried. Model 1 adjusted for age, sex, region, education, types of health insurance, history of stroke, hypertension, diabetes, dyslipidaemia, atrial fibrillation, coronary heart disease, pneumonia, current or previous smoking, moderate or heavy alcohol, body mass index (BMI) at admission, baseline NIHSS and educational level. Model 2 adjusted for model 1 covariates and included occupational class and personal income level.

**Supplementary Figure 1:**

**
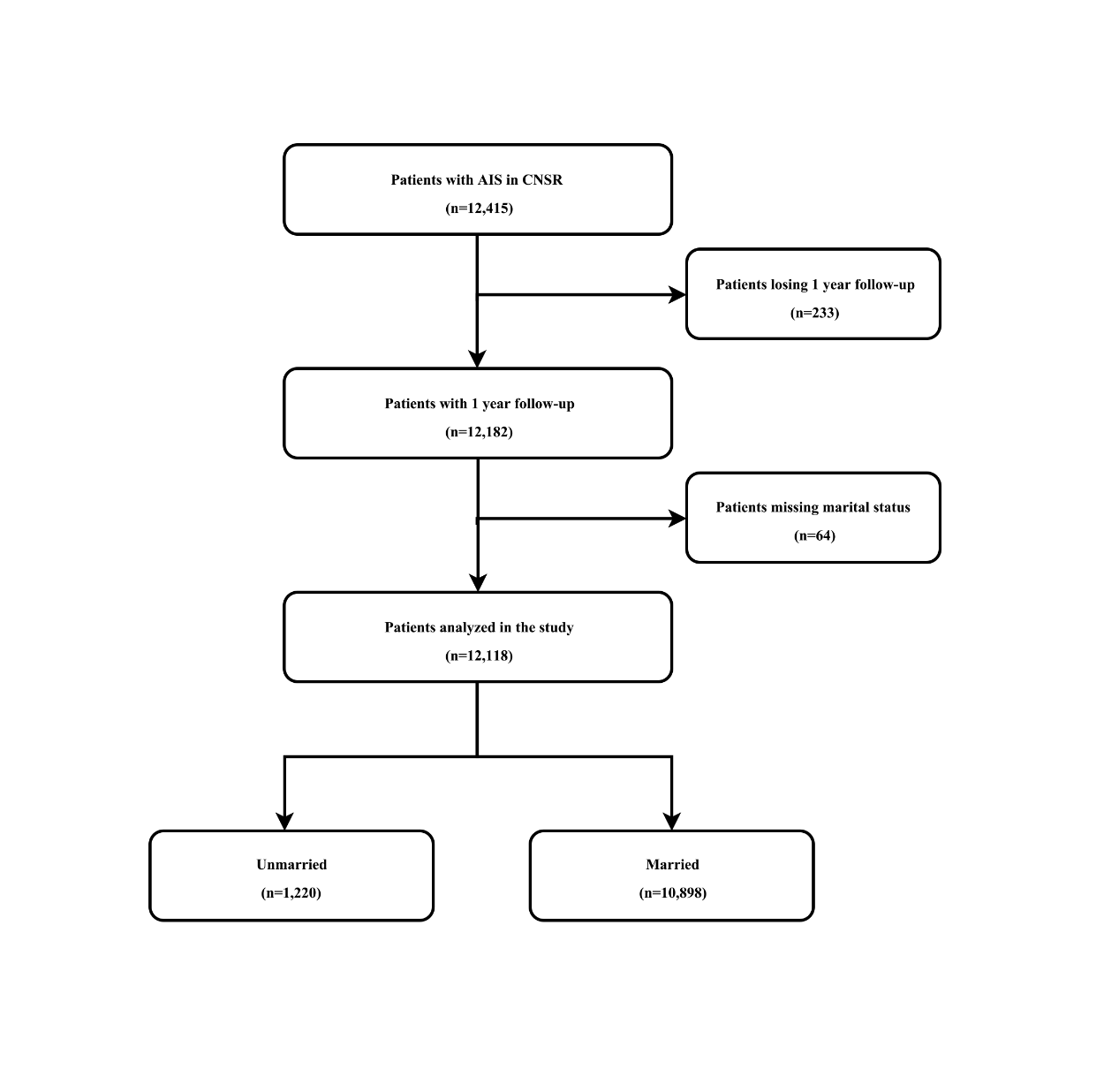
**

Flow chart showing the patients selection from China National Stroke Registry. AIS, acute ischemic stroke; unmarried includes widowed, divorced and never married; married includes continuously married and remarried; CNSR, the China National Stroke Registry.
